# Too much medicine: not enough trust?

**DOI:** 10.1136/medethics-2018-104866

**Published:** 2018-10-26

**Authors:** Zoë Fritz, Richard Holton

**Affiliations:** 1 The Healthcare Improvement Studies Institute (THIS Institute), University of Cambridge, Cambridge, UK; 2 The Division of Health Sciences, Warwick Medical School, Coventry, UK; 3 Faculty of Philosophy, University of Cambridge, Cambridge, UK

**Keywords:** allocation of healthcare resources, autonomy, clinical ethics, philosophy of medicine, truth disclosure

## Abstract

As many studies around the theme of ‘too much medicine’ attest, investigations are being ordered with increasing frequency; similarly the threshold for providing treatment has lowered. Our contention is that trust (or lack of it) is a significant factor in influencing this, and that understanding the relationship between trust and investigations and treatments will help clinicians and policymakers ensure ethical decisions are more consistently made. Drawing on the philosophical literature, we investigate the nature of trust in the patient–doctor relationship, arguing that at its core it involves a transfer of discretion. We show that there is substantial empirical support for the idea that more trust will reduce the problem of too much medicine. We then investigate ways in which trust can be built, concentrating on issues of questioning, of acknowledging uncertainty and of shouldering responsibility for it. We argue that offering investigations or treatments as a way of generating trust may itself be an untrustworthy way of proceeding, and that healthcare systems should provide the institutional support for facilitating continuity, questioning and the entrusting of uncertainty.

## Introduction

There is a mounting body of evidence that investigations are being ordered with increasing frequency; similarly the threshold for providing treatment has lowered.^1–3^ When these investigations and treatments are actively harmful to patients then we are inflicting ‘Too Much Medicine’.

We start with a case study.

A 40-year-old man attends hospital with chest pain. He is worried that he might have a clot on his lung; a friend of his died last year from such a condition. His pain is achy, and came on over a few days. He has a cough, but is not bringing up any sputum or blood. He has no breathlessness. The doctor’s examination finds nothing amiss except a mildly raised pulse and temperature. Blood results are suggestive of infection. The doctor is confident that his pain is caused by a mild chest infection, and not from a pulmonary embolism (PE)—the clot on the lung that the patient was fearing.

The ‘gold standard’ to exclude a PE is a CT pulmonary angiogram (CTPA): dye is injected into the veins so that the pulmonary arteries can be seen clearly and any clot identified. The patient is exposed to radiation—the equivalent of about 5 years’ background exposure. It is known that this is linked to increased cancer risk, a risk that increases the younger the patient: a 40 year-old is more at risk than a 60 year-old.^4^

The doctor is thus faced with a choice. She can reassure the patient that, in her clinical judgment, he does not have a PE; or she can order a test which will prove that he does not, but which increases his lifetime cancer risk. Or she can involve her patient in this decision of whether to do the test. While this might be lauded as respecting the patient’s autonomy, it could also be seen as an abrogation of the physician’s responsibility: a responsibility to weigh the risks and benefits and take on the anxiety associated with the uncertainty inherent in medicine, as issue to which we will return.

**Figure 1:**
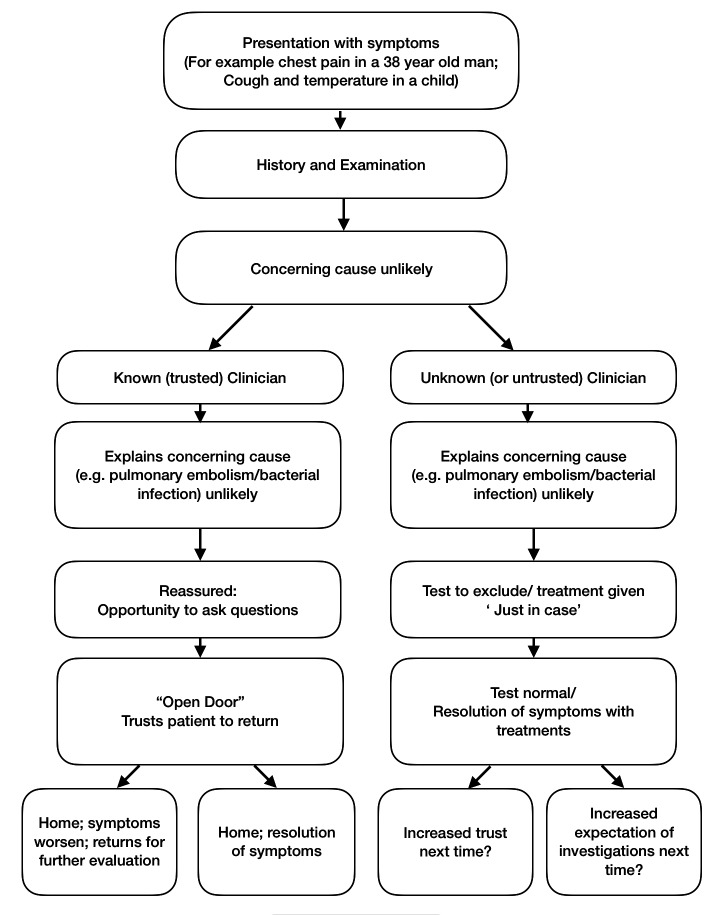
Possible routes (of investigation or non-investigation, treatment or non-treatment) that doctors and patients might take in the the presence and absence of trust.

Modifying this case to one where a patient attends with a desire for antibiotics, we get a parallel situation involving treatment rather than investigation (see figure 1). The doctor does not believe the patient has a significant bacterial chest infection; she thinks the symptoms will resolve on their own. If her clinical judgment is trusted, she can reassure him, and he will go home without medication. If, however, trust is lacking, then the doctor may choose to provide a prescription of antibiotics ‘to be on the safe side’.

These choices—and tens of thousands like them made daily—present several conflicts: between the potential harm of the investigation or treatment, and the potential harm of failing to diagnose a serious condition; between the use of resources for the potential benefit of an individual, and their use for the more certain benefit of others; and between the doctor’s responsibility to make a decision based on clinical expertise, and the patient’s right to take part in shared decision-making.

Our contention is that trust is a significant factor in influencing these choices, and that understanding the relationship between trust and investigations and treatments will help clinicians and policymakers ensure ethical decisions are more consistently made.

If, in our first case, the patient trusts the doctor, she is likely to be able to reassure him without recourse to the proof of a scan. This reassurance might take the form of explaining her reasoning, including what she believes is the most probable cause for his symptoms, being open about the remaining level of uncertainty and encouraging him to get in touch if his symptoms change—what medics call ‘leaving the door open’.

If he does not trust her, or if she doubts that he does, she is more likely to feel compelled to request the CTPA even if she does not think it is truly indicated. Various motivations might underlie such a request in this context: (1) a desire to reassure him that she feels cannot be achieved without further evidence, especially evidence provided by impressive technology; (2) a desire to end the consultation quickly due to time pressures (easier to order a scan than take time explaining to a sceptical patient why one is not needed); (3) a desire to protect against potential litigation; and (4) a desire to create trust in her clinical abilities for future consultations. As the doctor might say: ‘I don’t think you have a PE, but, to be on the safe side, we’ll order a test, and then we’ll both know for sure.’

Trust is also important in the opposite direction. If the doctor trusts the patient she will think both that he is accurately reporting his symptoms, and, perhaps more importantly, that if they were to change in a worrying way, he would come back. But if she lacks that confidence, again she is more likely to order tests.

Our aim here is to provide a synthesis of discussions of the nature of trust, drawing on both philosophical and medical literature; to investigate the empirical grounds for thinking that its lack may lead to overinvestigation and overtreatment; and finally to suggest some ways that clinicians and patients can work together to establish well-placed trust in the limited time they have.

## The nature of trust

Attempts to characterise the nature of trust have proved controversial.^5^ Here we try to remain in largely uncontentious territory above the fray. Trusting someone involves both a behaviour—a readiness to rely on them—and an attitude. To see the difference observe that, if there is no good alternative, we might rely on someone without trusting them; mere reliance thus falls short of trust.^6^ When we trust someone we assume more: in the case of a doctor, we assume that they have our best interests at heart. Clearly, though, the reliant behaviour and the attitude interact: often we will rely *because* we trust. Trust, then, can be instrumentally valuable in encouraging reliant behaviour, but it is not just that. If it is well founded we value trust for itself and for the other benefits it brings: the patient who trusts their doctor is likely to be better off—more reassured, more confident, better able to cope with their illness^7^—than a patient who does not. Conversely, if trust is not well founded it can be profoundly harmful. If we find our trust has been let down, the reaction will not be simple disappointment, but a sense of betrayal.^8^ So, quite rightly, we tend to be cautious about whom we trust; we should only trust the truly trustworthy.^9^ A trusting relationship will typically be built up over time as we gain evidence that our trust is well placed.

This last point brings us to a tension: while checking up on a person we are to trust can look like a sensible precaution, it can itself be undermining of trust. If we are constantly questioning and checking can we really be said to trust? Since trust builds further trust, and a lack of trust destroys it, a trusting relationship can scarcely be expected to flourish if one party is discovered to be checking on the other.^8 10^

Doctor–patient trust is marked by asymmetry. The doctor typically has knowledge that the patient lacks, and the power to order investigations and treatments. There is asymmetry in the other direction too: the patient has knowledge about their symptoms, and their family and social history. And they also have their own power: to keep the doctor informed of new developments, to follow a treatment plan, to seek a second opinion, to withdraw altogether and seek help elsewhere.

A trusting relationship between doctor and patient typically involves the granting of discretion in the light of these asymmetries. Our focus is on the discretion granted to the doctor. This may simply involve the discretion to implement a treatment plan that has already agreed on, but it may go further than that. It may include giving the doctor discretion to weigh evidence and to make decisions on the patient’s behalf when they are in no position to make them themselves, perhaps when they do not really understand what is at stake. Uncertainty adds further scope for discretion. Diagnoses are rarely certain; treatment outcomes even less so. Some patients are happy to cope with uncertainty, even if presented in the most abstract statistical ways. But for many, uncertainty can be itself unwelcome, something else that they would rather trust the doctor to look after.

So we can see both why trust is desirable in a medical context, and why it might be hard to achieve. In a world of anonymous emergency departments, team practices with multiple handovers and highly mobile patients, trusting relationships are not built as easily as they might be with a traditional family doctor. The very practices of questioning that may be needed to establish whether trust would be well founded may work to undermine it. And the uncertain information that doctors feel able to give may be not what their patients want from a figure they do not yet trust. Small wonder if doctors order tests and offer treatments as a substitute.

We think though that the situation is far from hopeless: there is much that can be done to establish well-founded trust once the problems are properly understood. The very questioning that can seem to be undermining of trust can be channelled to help build it. First though we examine the empirical evidence.

## Empirical studies: does too little trust lead to too much treatment?

Is there any concrete evidence that patients receive more investigations, and more treatment, if they do not have a trusting relationship with their doctor? There is very little research targeting this question directly, but a number of studies are relevant.

### Continuity, trust and overinvestigation

There is evidence that continuity allows time for trust to be built. A survey of more than 1000 British and American patients suggested trust builds over repeated encounters.^11^ Semistructured interviews with 20 patients in the UK^12^ suggested that trust at the initial encounter relied on what David Mechanic calls ‘institutional trust’,^13^ that is, trust in the institution independently of the doctor, which is sufficient for simple interactions. So-called ‘swift trust’^14^ could then be established by ‘effective communication and development of common understanding during the consultation…[but this was] fragile and easily undermined’; finally, ‘repeated interactions not only allowed patients to validate the GP as trustworthy but also enabled patients to build their own reciprocal reputation with their GP.’^14^ This paper suggests that it is not only important for the general practitioner to trust the patient, but for the patient to recognise that they are trusted. One participant said: ‘You know he understands that if you’re complaining about something, you’ve jolly well got something and you’re not sort of making it up or wasting his time.’

Increased continuity of care has, in turn, been shown to be associated with a decrease in overuse of procedures. A retrospective observation of which procedures were undertaken in more than a million randomly selected Medicare patients revealed that for each 0.1 increase in the continuity score, patients had 0.93 times the odds of receiving overused (and probably unneeded) procedures (such as laryngoscopy for sinusitis or MRI for mild traumatic brain injury) than those with lower scores (95% CI 0.93 to 0.94).^15^ The authors highlight several contributing factors; foremost among them was trust. (Communication^16^ and patient satisfaction^17^ are the other two which may not be independent: plausibly good communication leads to trust, and higher trust leads to greater satisfaction.)

A retrospective study of 230 470 patients found that higher continuity of care was associated with fewer hospital admissions for conditions that could be treated as an outpatient.^18^ While this could be attributed to familiarity with a patient (a doctor who has not met a chronically breathless patient before might be concerned by their symptoms and seek a hospital assessment; in contrast a doctor who has been trying to manage their condition for some time might recognise the breathlessness as ‘normal for them’), the authors of this paper suggest that, among the contributing factors, ‘continuity of care might also promote a more effective and trusting relationship between patients and doctors.’ A trusted doctor might be more able to reassure a patient that attendance into hospital for further investigations was not needed.

### Trust and overtreatment

A relationship between continuity of care, the establishment of secure trust and a change in the threshold for investigation is therefore possible; is there also evidence for a relationship between trust and treatments given?

Jabaaij and colleagues conducted a retrospective cohort study of more than 10 000 patients in 104 general practices in the Netherlands.^19^ Newly enlisted patients were matched for age, sex and health insurance with those who had been enlisted for more than a year. Newer patients were found to have a higher probability of receiving a prescription for antibiotics, and higher overall use of resources. The authors ask whether this may be due to ‘the general practitioner behaving more defensively when treating patients for the first time.’ An alternative interpretation is that the doctor is prescribing antibiotics as a mechanism for building trust with a new patient, to demonstrate that they can be relied on to take the patient’s complaint seriously.

Physician perception of a patient’s desire for treatment is perhaps more important than the patient’s expressed needs: a systematic review of the factors potentially implicated in unnecessary antibiotic use in respiratory tract infections revealed that the only non-clinical factor was physician perception of a patient’s desire for antibiotics; an explicit request for antibiotics was not associated with higher likelihood of having them prescribed.^20^ We postulate that a patient–doctor relationship with secure trust would diminish the possibility of a physician second guessing that their patient desired antibiotics. Supporting this idea, a small qualitative study of 16 Icelandic doctors’ reasons for ‘non-pharmacological’ prescribing of antibiotics suggested that a significant reason was: ‘an unstable doctor-patient relationship due to lack of continuity of care’^21^ and a large qualitative study of four nations suggested that trust was a major factor in parents accepting physician non-prescribing. Combined, these studies suggest that new, non-secure patient–doctor relationships are more prone to overprescribing than those in which continuity has allowed trust to develop.

### Trust and choosing less medicine

One condition associated with increasing invasive procedures is prostate cancer.^22^ Since prostate cancer is often slow growing, men can choose to have ‘active surveillance’ where the prostate-specific antigen (PSA) is checked regularly to make sure the disease does not progress. Alternative approaches, including surgery or radiotherapy, are ‘definitive’ (they remove the uncertainty of whether the cancer will progress) but carry risks of significant side effects.

A US study found that patients who trusted their doctors were more likely to follow their advice to have active surveillance instead of surgery.^23^ A higher level of trust also made it more likely that the doctor would recommend active surveillance. What is happening here? The perception of some patients was that invasive treatments were recommended from a desire for profit, and this may have undermined trust. Or it is possible that patients prefer to have fewer invasive procedures, and so trust the doctor who advises such a course, or than the trust is built because a particular course is recommended and followed.

To disentangle this, a prospective study is needed, assessing first baseline patient preference for invasive treatment or active surveillance; and baseline trust in the physician. Then it needs to assess the level of trust, and the decision made, following the physician’s recommendation. Nevertheless, it is interesting to consider that doctors may be carrying out investigations or giving treatments in an effort to build trust when, in this example, trust may be more reliably built by expressing a recommendation for ‘watchful waiting’.

## Towards a solution: continuity and questioning

A first thought is that, if we need to build trusting relationships, and these take time, then continuity of care is essential. It is not good enough to ensure that records are passed on. Trust is built with individuals, and so the same individuals need to be involved with a patient’s care.

What though in cases where this is not possible? Here we want to go back to the tension flagged at the beginning between trust and questioning. How deep does that go? There are certainly personal relationships where questioning is inappropriate. Quizzing a partner on everything they have done in an attempt to establish their fidelity is no way to establish trust. But the medical context is different, and seeing why helps us to see how questioning can actually help to establish trust here.

Questioning in a medical relationship is *needed*. It is needed for the simple transfer of information, from patient to doctor, and from doctor to patient. Asking questions—and receiving answers—diminishes the clinical information imbalance between doctor and patient. Less obviously, it is needed to establish the form of the relationship that both sides are happy with: the amount of information that is wanted, the ways in which it is to be imparted, the degree to which discretion is to be transferred and over which topics.

Some patients are comfortable with uncertainty, and want, as far as possible, to make their own decisions about treatment and any associated risks. But many are not. Consider the questions that are often asked even when the likelihoods and risks have been presented: ‘But do *you* think I need the scan?’ ‘Should I worry about that?’ ‘What would you recommend?’ ‘What would you do if you were in my shoes?’ These are not requests for more information about the odds. Instead they indicate a readiness to transfer some discretion to the doctor: perhaps discretion to take decisions, but equally discretion to mark out the live possibilities within which decisions are to be taken. This is not to give the doctor *carte blanche*. And it is certainly not to ask the doctor to stop monitoring the possibilities. On the contrary: if the doctor advises not having a scan, and that advice is accepted, the expectation is that the doctor will be keeping track of things so that if a scan comes to be indicated it will be ordered.

In an atmosphere of trust, where the patient understands what is happening, giving discretion in this way does not infringe their autonomy: by removing some of the weight of uncertainty, it can enable it to function more effectively.^24^ The doctor can seek to understand the patient’s preferences in terms of the level of uncertainty they are comfortable with, as well as the levels of investigations and treatments the patient prefers.^25^ It is only in the give and take of questioning—questioning that reveals the concerns of both doctor and patient—that an appropriate balance can be established.

## Conclusion

What happens when the clinician takes the other path and provides an extra test or treatment in the absence of trust? What effect will this have on the degree of trust in future encounter with this physician? It is possible that providing an investigation or treatment once raises expectations that this is what will always happen. Conversely, it is possible that, having had the reassurances that a test would probably be normal and then having it proved to be so, the patient will have more trust in the physician and thus be more willing to accept verbal reassurances next time (see dotted lines in figure 1). Some empirical investigation here would be useful. But even if it proves that unnecessary testing is a way of building trust, we want to resist the idea that this is a legitimate way of proceeding. Engendering trust in this way is itself untrustworthy behaviour. To expose a patient to an unnecessary risk, such as the radiation from a CT scan or the side effects from antibiotics, is to cause them harm. The potential secondary gain of building trust for future encounters is undermined by the dishonesty which accompanies this motive: it is not trustworthy behaviour to manipulate a patient into trusting you by preforming an unnecessary test.

An alternative, more trustworthy (but slower) way of building trust is for physicians to encourage questioning, to be open about the uncertainties and to take on responsibility for those uncertainties. Doing this is a necessary but insufficient step: health systems would need to support physicians in providing opportunities for follow-up and continuity in the absence of overtesting. Such follow-up could be planned or ‘SOS’; telephone or email communication may be better than the traditional outpatient visit. This would provide the opportunity for more questions, and would offer assurances to the patient and to the doctor: assurance that they had made the right decision in not providing a test or treatment that would deliver too much medicine.
